# Is Central Corneal Thickness a reliable independent factor in decision-making regarding the management of patients with high IOP?

**Published:** 2020

**Authors:** Georgiana Camburu, Mihail Zemba, Victor Lorin Purcărea

**Affiliations:** * “Carol Davila” University of Medicine and Pharmacy, Bucharest, Romania

**Keywords:** Central Corneal Thickness, GAT IOP, adjusted IOP

## Abstract

As time passes, we discover that things that were supposedly considered to be well known are in fact not as they seemed. Maybe the process is like this because we try to simplify our daily practice, but by doing so, don’t we tend to trespass the whole picture? However, what happens if these factors affect our decision-making?

Clinicians should incorporate CCT into the thinking process and should not focus on “corrected IOPs”, because, besides CCT, there are many factors of corneal biomechanics that affect IOP.

As time passes, we discover that things that were supposedly considered to be well known, are in fact not as they seemed. Maybe the process is like this because we try to simplify our daily practice, but by doing so, don’t we tend to trespass the whole picture? However, what happens if these factors have an impact on our decision-making?

CCT has been considered a useful tool in measuring IOP for long time. Various formulas were made to adjust IOP. It is advised that normal CCT is 550 microns. If so, why is Goldmann Applanation Tonometer (GAT) considered the Gold Standard [**1**] and why can it give accurate results, even if it does not take into consideration CCT?

The Goldmann device was invented in 1950 [**2**] and is a tonometer described by the Imbert-Fick principle [**3**]. In an ideal sphere with dry thin walls, the internal pressure is equal with the force divided by the area of the flattening. Goldman stated that using a 3.06 mm diameter, the resistance of the cornea [**3**] during the flattening is compensated by the tear film meniscus capillary attraction [**4**,**5**].

Because many clinicians were measuring the CCT during every examination mainly to adjust the IOP that was taken by using GAT, The American Academy of Ophthalmology conducted a study which revealed that the use of CCT in adjusting IOP did not predict POAG. The report stated clearly that CCT still remains a significant component of complete ocular examination, as an independent predictive factor for POAG [**6**]. 

In a study made by Brandt et al., a re-analysis of the OHTS evaluated the prediction developing of POAG. They compared the prediction accuracy of POAG for un/ adjusted IOP with five different formulas [**5**]. After examining the data of 1433 participants, the conclusion was that for calculating the risk of developing POAG in patients with ocular hypertension is accurate to use IOP and CCT obtained and not to apply correction formulas [**5**]. 

After reviewing the results of Brandts et al., Dr. Medeiros suggested that instead of using IOP formulas, clinicians should better incorporate risk information as provided, and regarding the legitimacy of that CCT as a true independent glaucoma risk factor, he considered that further investigations are required [**5**].

In a study that aimed to evaluate if CCT could be a useful tool for the disease progression prognostic in ocular hypertensives, the author recommended that measuring CCT is advisable when the clinical finding does not correlate with the IOP token by the applanation technique [**7**].

Dr. Elliot Kirsten conducted a study trying to determine the validity of Ehler monogram in adjusting GAT IOPs. Ehlers monogram is widely used. He reveled that using the monogram in correcting the error made by omission of the CCT will increase the error instead of decreasing it [**8**].

In the article entitled “Corneal thickness: It's time we all get rid of the correction factor from the glaucoma equation”, Dr. Al Busadi stated that clinicians should incorporate CCT into the thinking process and should not focus on “corrected IOPs”, because there are many factors, besides CCT, that have an impact on IOP such as: corneal hydration, connective tissue composition, and bio elasticity [**8**].

 In fact, there are other biomechanical proprieties of the cornea that can affect IOP. Luce [**9**] was the first to introduce the term “Corneal Hysteresis” as an indicator of corneal biomechanics. Congdon [**10**] stated a lower Hysteresis, and not CCT, associating progression of visual fields defects of glaucoma patients. 

## Is Corneal Hysteresis a more reliable factor?

Dr. Pillai believes that CCT correction formulas provide less accurate IOP values and, since the cornea and the optic nerve are continuous structures, she considers that corneal hysteresis can be corelated with the ability of the optic nerve to tolerate intraocular pressure [**11**]. 

## Should GAT IOP measurements be corrected taking into consideration the corneal biomechanics properties?

Two hundred eighty-nine patients were subjected to IOP measurements using both PDCT and GAT. PDCT asserts that the IOP obtained is independent of the corneal properties. This study [**12**] conducted by dr. Park showed that there was a weak correlation between PDCT and GAT, and worse for GAT CCT adjusted, in patients with considered thick cornea. They suggested that adjusting GAT IOP in patients with increased corneal thickness may underestimate IOP, having as a result a delaying in starting the treatment [**13**]. A possible explanation was offered in the article entitled “Data do not support adjusting tonometry for central corneal thickness”. The author stated that “CCT is an important component of error in Goldmann, but it is almost certain that other factors, such as the viscoelastic properties of the cornea, minimize the influence of central corneal thickness on tonometry [**14**,**16**].

Dr. Ahmed Elshiekh, University of Dundee, Scotland, affirmed that GAT is difficult to correct and is affected by corneal stiffness [**14**], compound of thickness, curvature and biomechanical properties of the corneal tissue. A correction just for thickness is inadequate because it represents an incomplete approach. All the parameters were evaluated individually, but there is no guidance on how to combine these effects, as “they are all at play at the same time” [**8**]. 

## …so, which is the best approach?

CCT should be measured every 5 years because the value also decreases in time. Moreover, there are other factors that affect CCT measurements, such as prostaglandins, topical dorzolamide, topical beta-blockers, estrogen levels during ovulation and pregnancy, diabetes [**15**,**17**]. 

Dr. Steven R. Sarkisian Jr. takes into account the pressure and CCT and states that unless CCT is > 600 µm or < 500 µm, it does not let it affect his assessment [**13**,**18**].

A conclusion of Dr. Adam Reynolds is that we cannot build an accurate algorithm in adjusting IOP based on CCT, because IOP measurements have a high level of variability and we do not yet understand many of those variables [**13**,**18**]. 
